# Better survival and prognosis in SCLC survivors after combined second primary malignancies: A SEER database-based study

**DOI:** 10.1097/MD.0000000000032772

**Published:** 2023-02-10

**Authors:** Silin Wang, Sheng Hu, Shengfei Huang, Lang Su, Qiang Guo, Bo Wu, Jiayue Ye, Deyuan Zhang, Yang Zhang, Wenxiong Zhang, Yiping Wei

**Affiliations:** a Department of Thoracic Surgery, The Second Affiliated Hospital of Nanchang University, Nanchang, China; b Department of Medical Oncology, The First Affiliated Hospital of Nanchang University, Nanchang, China.

**Keywords:** SCLC, second primary malignant cancer, SEER, SPM, survival time

## Abstract

With recent advances in treatment modalities, the survival time for patients with small cell lung cancer (SCLC) has increased, along with the likelihood of recurrence of a second primary tumor. However, patient treatment options and prognosis remain uncertain. This research evaluated the survival rates of patients with SCLC with a second malignancy, aiming to provide new insights and statistics on whether to proceed with more active therapy. SCLC patients were selected based on the Surveillance, Epidemiology, and End Results (SEER) database, updated on April 15, 2021. We defined those with SCLC followed by other cancers (1st of 2 or more primaries) in the sequence number as S-second primary malignant cancer (S-SPM). Those who had other cancers followed by SCLC (2nd of 2 or more primaries) were defined as OC-SCLC. We performed Kaplan–Meier survival analysis, life table analysis, univariate analysis, stratified analysis, and multiple regression analysis of patient data. We considered the difference statistically meaningful at *P* < .05. After selection, data for 88,448 participants from the SEER database was included in our analysis. The mean survival time for patients with S-SPM was 69.349 months (95% confidence interval [CI]: 65.939, 72.759), and the medium duration of survival was 34 months (95% CI: 29.900, 38.100). Univariate analysis showed that for overall survival, the hazard ratio (HR) of S-SPM was 0.367 (95% CI: 0.351, 0.383), which was 0.633 lower than that of patients with solitary SCLC and 0.606 lower than that of patients with OC-SCLC. For cancer-specific survival (CSS), the HR of S-SPM was 0.285 (95% CI: 0.271, 0.301), which was 0.715 lower than for patients with solitary SCLC and 0.608 lower than that for patients with OC-SCLC. Multiple regression analysis showed that the HR values of S-SPM were lower than those of patients with single SCLC and those with OC-SCLC, before and after adjustment for variables. Kaplan–Meier survival curves showed that patients with S-SPM had significantly better survival times than the other groups. The survival time and prognosis of patients with S-SPM were clearly superior to those with single SCLC and OC-SCLC.

## 1. Introduction

Lung cancer remains the world’s deadliest disease, accounting for 1 quarter of deaths from all cancers. Histologically, pulmonary cancers are separated into 2 major categories: non-small cell lung cancer and small cell lung cancer (SCLC). SCLC represents an estimated 13% to 15% of lung cancer.^[1,2]^ SCLC is notorious for its high level of aggressiveness. SCLC exhibits a high frequency of variants in both tumor suppressors and oncogenes^[3,4]^ and is highly metastatic,^[5–7]^ with nearly 70% of patients already having metastasis to lymph nodes and to distant sites when diagnosed. In 2013, SCLC was nominated as “persistent cancer” by the National Cancer Institute.^[8]^ These observations underscore the poor prognosis of SCLC. Treatment of SCLC has been less than satisfactory. With advances in the treatment modalities of SCLC in recent years, the survival time of patients with SCLC has been extended, while the probability these patients developing a second primary tumor during their survival has increased.^[9]^ This poses new challenges for clinicians.

Second primary malignant cancer (SPM) refers to the occurrence in a single or multiple organs of the same individual, developing after the first primary malignancy, independent of the first primary malignancy, rather than metastasis or recurrence.^[10–12]^ SPMs appear essentially as a result of acquired genetic mutations or inheritance and might occur very late or within a short period of time following therapy for the first primary tumor, possibly indicating potential genetic or immune deficiency of the patient, genetic damage related to therapy, or environmental contact with carcinogens.^[13–15]^

As the number of patients with SCLC with comorbid second primary tumors continues to increase, there is growing concern about this phenomenon. However, few studies have reported patients with SCLC with a concurrent second primary tumor during survival, which negatively affects the development of treatment plans for patients with second cancers. Therefore, further study of this phenomenon is necessary. The present study analyzed the latest SCLC statistics contained in the Surveillance, Epidemiology, and End Results (SEER) database for 88,448 patients with SCLC. SEER is the definitive U.S. cancer statistics database, which captures information on the incidence, fatality rates, and disease status among millions of patients with malignancies in certain U.S. states and counties. The goals of this research were to compare the clinical results of patients with SCLC complicated by a second primary tumor during survival with those of patients with SCLC alone and patients with other cancers with concurrent SCLC during their survival, and to assess and forecast the survival of patients with SCLC connected with a second primary tumor, providing new data and insights about whether to provide aggressive therapy.

## 2. Methods

### 2.1. Data source

Using the updated SEER data from April 15, 2021, we selected 91,332 SCLC cases. Roughly 27.8% for the U.S. population is captured in the SEER database (based on 2010 subpopulation statistics). We selected 22 entries, including Age recode with <1 year old, Chemotherapy recode (yes, no/unknown), Diagnostic confirmation, Grade (through 2017), ICD-O-3 Hist/behav, Laterality, Marital status at diagnosis, Origin recode NHIA (Hispanic, Non-Hisp), PRCDA 2017, Patient ID, Radiation recode, Regional nodes positive (1988+), Race recode (White, Black, Other), Survival months, Sequence number, Sex, Summary stage 2000 (1998–2017), Site specific surgery (1973–1997 varying detail by year and site), SEER cause-specific death classification, Total number of in situ/malignant tumors for each patient, Vital status recode (study cutoff used), and Year of diagnosis.

### 2.2. Data processing

**Figure 1.**
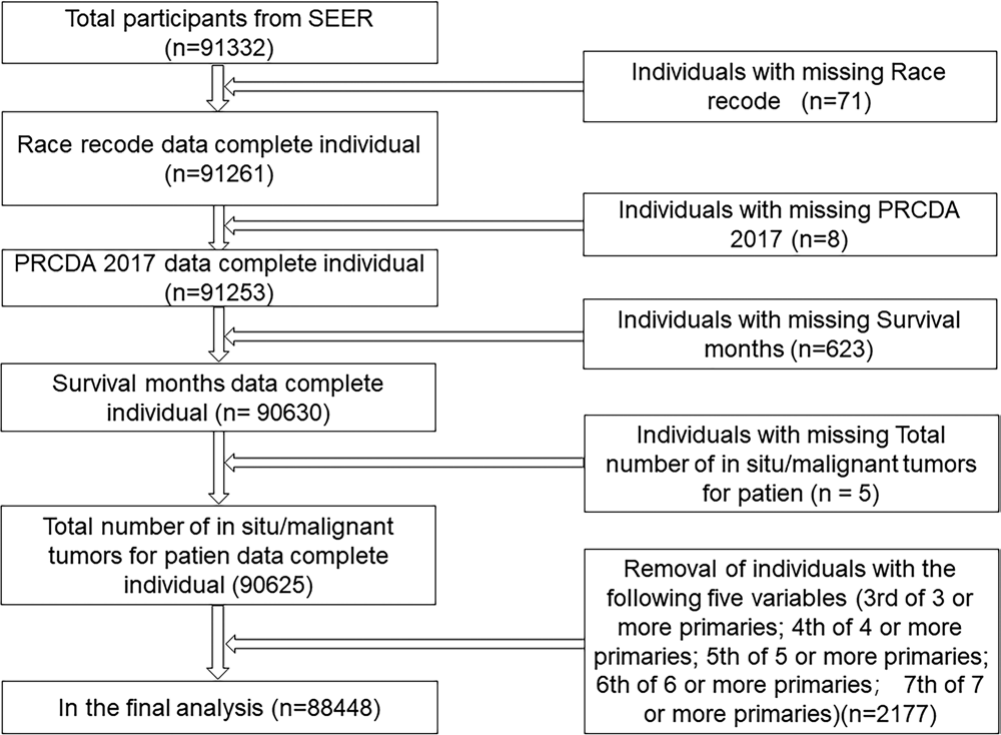
Data screening chart for patients with SCLC. SCLC = small cell lung cancer.

We removed 707 missing values from the different variables and then removed 2177 items from the following 5 variables with less data (3rd of 3 or more primaries; 4th of 4 or more primaries; 5th of 5 or more primaries; 6th of 6 or more primaries; 7th of 7 or more primaries). A total of 88,448 individuals with SCLC were ultimately enrolled in our study (Fig. 1). Individual entries were integrated and grouped, and for a more concise description of our study, we defined those with SCLC followed by other cancers (1st of 2 or more primaries) in the sequence number as S-SPM. Those who had other cancers (OC) followed by SCLC (2nd of 2 or more primaries) were defined as OC-SCLC.

### 2.3. Statistical analysis

The statistical analysis was performed using statistics as a function of frequency and using SPSS v.24 (IBM Corp., Armonk, NY). Trends in survival time were plotted for different subgroups using GraphPad Prism 8 (GraphPad Inc., La Jolla, CA). Data were analyzed using the statistical package R version 3.6.3^[16]^ (R Foundation for Statistical Computing. URL https://www.R-project.org/) and Empower Stats^[17]^ (https://www.empowerstats.net/cn/, X&Y solutions Inc. Boston, MA). To investigate the differences in patient prognosis, we carried out univariate analysis, Kaplan–Meier survival analysis, life table analysis, and stratified analysis of patient data. We considered significant differences statistically at *P* < .05. Furthermore, we performed a multiple regression analysis, in which the sequence number was used as the exposure variable, and status and cause of death recoded were used as the outcome variables. Non-adjusted meant without any adjustment. The adjust I model was adjusted by age, race, and sex. The adjust II model was adjusted by age, chemotherapy, cancer-directed surgery, diagnostic confirmation, grade, laterality, marital status, origin, PRCDA, race, radiation, regional nodes positive, sex, and summary stage.

### 2.4. Ethics declaration

The Ethical Review Committee of the National Center for Health Statistics approved all SEER protocols and obtained written informed consent from all participants.

## 3. Result

After screening, a total of 88,448 participants in the SEER database were included in our analysis, of whom 55.046% were male, 10.053% were over 80 years of age, 97.251% were Non-Spanish-Hispanic-Latino, 38.195% had undifferentiated SCLC, and 32.760% had distant metastases. Among the patients, 45.740% had a history of radiation therapy, 70.562% had a history of chemotherapy, 2.967% had a history of surgery, 55.586% had a companion, and 86.958% of deaths were directly attributable to SCLC. Baseline data for the rest of the population are detailed in Table 1.

**Table 1:** Baseline characteristics of participants (*N* = 88448).

Sequence number	*N* (%)	One primary only	1st of 2 or more primaries	2nd of 2 or more primaries	*P* value
Yr of diagnosis		1996.293 ± 12.073	1996.759 ± 11.449	2000.554 ± 11.372	<.001
Sex (%)					<.001
Female	39761 (44.954%)	44.1	45.1	50.3	
Male	48687 (55.046%)	55.9	54.9	49.7	
Age (%)					<.001
<60 yrs	21457 (24.259%)	26.0	26.6	12.8	
60–69 yrs	31216 (35.293%)	35.8	39.9	31.4	
70–79 yrs	26883 (30.394%)	29.0	26.3	39.8	
80+ yrs	8892 (10.053%)	9.2	7.3	16.0	
Race (%)					<.001
White	77417 (87.528%)	87.4	86.4	88.8	
Black	7102 (8.030%)	8.1	8.6	7.7	
Other	3929 (4.442%)	4.6	5.0	3.4	
Origin (%)					.244
Non-Spanish-Hispanic-Latino	86017 (97.251%)	97.2	97.1	97.5	
Spanish-Hispanic-Latino	2431 (2.749%)	2.8	2.9	2.5	
PRCDA (%)					.019
PRCDA	32639 (36.902%)	36.7	36.0	38.0	
Not PRCDA	55809 (63.098%)	63.3	64.0	62.0	
Grade (%)					<.001
Differentiated	5771 (6.525%)	6.4	8.4	7.1	
Undifferentiated	33783 (38.195%)	38.9	39.9	33.4	
Unknown	48894 (55.280%)	54.7	51.8	59.5	
Laterality (%)					<.001
Right-origin of primary	46622 (52.711%)	52.7	53.9	52.7	
Left-origin of primary	35330 (39.944%)	40.1	40.9	39.0	
Other	6496 (7.3444%)	7.3	5.2	8.3	
Diagnostic confirmation (%)					<.001
Positive histology	73238 (82.803%)	82.9	84.3	81.7	
Positive exfoliative cytology	13914 (15.731%)	15.6	14.9	16.9	
Positive other	1296 (1.465%)	1.5	0.7	1.4	
Summary stage (%)					<.001
Distant	28976 (32.760%)	32.1	20.2	39.3	
Regional	8325 (9.412%)	8.6	18.7	12.3	
Localized	2055 (2.323%)	1.9	6.6	4.2	
Unknown	49092 (55.504%)	57.4	54.5	44.2	
Radiation (%)					<.001
Yes	40456 (45.740%)	46.1	57.9	40.9	
No	46562 (52.643%)	52.2	40.0	57.7	
Unknown	1430 (1.617%)	1.6	2.1	1.4	
Chemotherapy (%)					<.001
Yes	62411 (70.562%)	70.8	78.9	67.4	
No	26037 (29.438%)	29.2	21.1	32.6	
Cancer-directed surgery (%)					<.001
Yes	2624 (2.967%)	2.9	7.0	2.6	
No	21790 (24.636%)	25.0	23.8	22.6	
Unknown	64034 (72.397%)	72.1	69.1	74.8	
Regional nodes positive (%)					<.001
Nodes negative	1266 (1.431%)	1.2	5.4	2.1	
Nodes positive	9722 (10.992%)	10.5	14.1	13.4	
Unknown	77460 (87.577%)	88.3	80.5	84.5	
Marital status (%)					<.001
Accompanied	49165 (55.586%)	55.7	60.4	54.2	
Alone	36452 (41.213%)	41.2	37.2	42.3	
Unknown	2831 (3.201%)	3.2	2.4	3.5	
Sequence number (%)					<.001
One primary only	73995 (83.659%)	100.0	0.0	0.0	
1st of 2 or more primaries	2418 (2.734%)	0.0	100.0	0.0	
2nd of 2 or more primaries	12035 (13.607%)	0.0	0.0	100.0	
Status (%)					<.001
Alive	3070 (3.471%)	3.1	9.7	4.4	
Dead	85378 (96.529%)	96.9	90.3	95.6	
Cause of death recoded (%)					<.001
Dead of cancer	76913 (86.958%)	11.1	40.4	19.4	
Alive or dead of other cause	11535 (13.042%)	88.9	59.6	80.6	

Note: Outcome variable: Status; Cause of death recoded. Exposure variable: Sequence number.

Univariate analysis of SCLC revealed that patients with S-SPM had significantly longer survival times than patients with SCLC only and those with OC-SCLCs. In terms of overall survival (OS), the hazard ratio (HR) for patients with S-SPM was 0.367 (95% confidence interval [CI]: 0.351, 0.383), a decrease of 0.633 compared with patients with SCLC alone. In terms of cancer-specific survival (CSS), the HR for patients with S-SPM was 0.285 (95% CI: 0.271, 0.301), a decrease of 0.715 compared with patients with SCLC alone. Both P values were less than 0.05. In patients with OC-SCLC, the HR was 0.973 (95% CI: 0.954, 0.993) for OS, an increase of 0.606 compared with that of patients with S-SPM, and 0.893 (95% CI: 0.874, 0.912) for CSS, an increase of 0.608 compared with that of patients with S-SPM, both had P values less than 0.05. The main covariates in this study were sex, age, race, origin, PRCDA, grade, laterality, diagnostic confirmation, summary stage, radiation, chemotherapy, cancer-directed surgery, regional nodes positive, and marital status. The HR values, 95% CIs and P values for the above covariates are detailed in Table 2. We also performed a stratified analysis, which confirmed that patients with S-SPM had a clearly longer survival duration than patients with SCLC only and those with OC-SCLC (see Table S1, http://links.lww.com/MD/I380, Supplemental Content, which illustrates the stratified analysis of SCLC).

**Table 2:** Univariate analysis of small cell lung cancer.

Exposure	Statistics	Status	*P* value	Cause of death recoded	*P* value
	*N* (%)	HR (95%CI)		HR (95%CI)	
Sequence number					
One primary only	73995 (83.659%)	Reference^[1]^		Reference^[1]^	
1st of 2 or more primaries	2418 (2.734%)	0.367 (0.351, 0.383)	<.0001	0.285 (0.271, 0.301)	<.0001
2nd of 2 or more primaries	12035 (13.607%)	0.973 (0.954, 0.993)	.0068	0.893 (0.874, 0.912)	<.0001
Yr of diagnosis	1996.885 ± 12.052	0.996 (0.995, 0.996)	<.0001	0.995 (0.995, 0.996)	<.0001
Yr of diagnosis Tertile					
Low	28201 (31.884%)	Reference^[1]^		Reference^[1]^	
Middle	28723 (32.474%)	0.898 (0.883, 0.913)	<.0001	0.900 (0.885, 0.916)	<.0001
High	31524 (35.641%)	0.891 (0.876, 0.906)	<.0001	0.886 (0.871, 0.902)	<.0001
Sex					
Female	39761 (44.954%)	Reference^[1]^		Reference^[1]^	
Male	48687 (55.046%)	1.190 (1.174, 1.206)	<.0001	1.187 (1.170, 1.204)	<.0001
Age					
<60 yrs	21457 (24.259%)	Reference^[1]^		Reference^[1]^	
60–69 yrs	31216 (35.293%)	1.189 (1.168, 1.211)	<.0001	1.147 (1.126, 1.168)	<.0001
70–79 yrs	26883 (30.394%)	1.472 (1.445, 1.499)	<.0001	1.394 (1.367, 1.421)	<.0001
80+ yrs	8892 (10.053%)	1.982 (1.932, 2.032)	<.0001	1.870 (1.821, 1.920)	<.0001
Race					
White	77417 (87.528%)	Reference^[1]^		Reference^[1]^	
Black	7102 (8.030%)	0.991 (0.967, 1.016)	.4839	0.976 (0.951, 1.002)	.0683
Other	3929 (4.442%)	0.925 (0.895, 0.956)	<.0001	0.908 (0.877, 0.941)	<.0001
Origin					
Non-Spanish-Hispanic-Latino	86017 (97.251%)	Reference^[1]^		Reference^[1]^	
Spanish-Hispanic-Latino	2431 (2.749%)	1.006 (0.965, 1.048)	.7896	0.998 (0.956, 1.043)	.9428
PRCDA					
PRCDA	32639 (36.902%)	Reference^[1]^		Reference^[1]^	
Not PRCDA	55809 (63.098%)	1.036 (1.022, 1.051)	<.0001	1.036 (1.021, 1.051)	<.0001
Grade					
Differentiated	5771 (6.525%)	Reference^[1]^		Reference^[1]^	
Undifferentiated	33783 (38.195%)	1.098 (1.067, 1.129)	<.0001	1.100 (1.068, 1.134)	<.0001
Unknown	48894 (55.280%)	1.124 (1.093, 1.155)	<.0001	1.124 (1.091, 1.157)	<.0001
Laterality					
Right-origin of primary	46622 (52.711%)	Reference^[1]^		Reference^[1]^	
Left-origin of primary	35330 (39.944%)	1.011 (0.997, 1.026)	.1187	1.010 (0.995, 1.025)	.2075
Other	6496 (7.344%)	1.283 (1.250, 1.318)	<.0001	1.278 (1.243, 1.314)	<.0001
Diagnostic confirmation					
Positive histology	73238 (82.803%)	Reference^[1]^		Reference^[1]^	
Positive exfoliative cytology	13914 (15.731%)	1.011 (0.992, 1.030)	.2482	0.998 (0.979, 1.018)	.8366
Positive other	1296 (1.465%)	1.343 (1.270, 1.420)	<.0001	1.346 (1.269, 1.426)	<.0001
Summary stage					
Distant	28976 (32.760%)	Reference^[1]^		Reference^[1]^	
Regional	8325 (9.412%)	0.488 (0.476, 0.501)	<.0001	0.463 (0.451, 0.476)	<.0001
Localized	2055 (2.323%)	0.391 (0.372, 0.410)	<.0001	0.338 (0.320, 0.356)	<.0001
Unknown	49092 (55.504%)	0.828 (0.816, 0.840)	<.0001	0.819 (0.806, 0.831)	<.0001
Radiation					
Yes	40456 (45.740%)	Reference^[1]^		Reference^[1]^	
No	46562 (52.643%)	1.661 (1.639, 1.684)	<.0001	1.658 (1.634, 1.682)	<.0001
Unknown	1430 (1.617%)	1.168 (1.107, 1.232)	<.0001	1.160 (1.096, 1.227)	<.0001
Chemotherapy					
Yes	62411 (70.562%)	Reference^[1]^		Reference^[1]^	
No	26037 (29.438%)	2.060 (2.029, 2.090)	<.0001	2.040 (2.009, 2.073)	<.0001
Cancer-directed surgery					
Yes	2624 (2.967%)	Reference^[1]^		Reference^[1]^	
No	21790 (24.636%)	1.687 (1.619, 1.758)	<.0001	1.821 (1.740, 1.906)	<.0001
Unknown	64034 (72.397%)	1.716 (1.649, 1.785)	<.0001	1.845 (1.766, 1.929)	<.0001
Regional nodes positive					
Nodes negative	1266 (1.431%)	Reference^[1]^		Reference^[1]^	
Nodes positive	9722 (10.992%)	1.858 (1.741, 1.983)	<.0001	2.082 (1.933, 2.242)	<.0001
Unknown	77460 (87.577%)	2.477 (2.328, 2.636)	<.0001	2.817 (2.624, 3.024)	<.0001
Marital status					
Accompanied	49165 (55.586%)	Reference^[1]^		Reference^[1]^	
Alone	36452 (41.213%)	1.107 (1.092, 1.123)	<.0001	1.094 (1.078, 1.110)	<.0001
Unknown	2831 (3.201%)	1.060 (1.019, 1.102)	.0034	1.035 (0.994, 1.079)	.0977

Note: Outcome variable: Status; Cause of death recoded. Exposure variable: Sequence number.

Multiple regression analysis demonstrated that patients with S-SPM had significantly better survival times than the other groups. In terms of OS, before adjustment for variables, the HR for patients with S-SPM was 0.367 (95% CI: 0.351, 0.383), which was 0.633 lower than that for patients with SCLC only and 0.606 lower than that for patients with OC-SCLC. In terms of CSS, the HR before adjustment for variables was 0.285 (95% CI: 0.271, 0.301) in patients with S-SPM, which was 0.715 lower than that in patients with SCLC only and 0.608 lower than in patients with OC-SCLC. With survival months as a time variable, sequence number as an exposure variable, and status and cause of death recoding as outcome variables, the results remained consistent with the non-adjusted results (Table 3).

**Table 3:** Multiple regression analysis of small cell lung cancer.

Exposure	Non-adjusted	*P* value	Adjust I	*P* value	Adjust II	*P* value
Status						
Sequence number						
One primary only	Reference^[1]^		Reference^[1]^		Reference^[1]^	
1st of 2 or more primaries	0.367 (0.351, 0.383)	<.00001	0.367 (0.351, 0.383)	<.00001	0.407 (0.390, 0.425)	<.00001
2nd of 2 or more primaries	0.973 (0.954, 0.993)	.00676	0.906 (0.888, 0.924)	<.00001	0.932 (0.914, 0.951)	<.00001
Cause of death recoded						
Sequence number						
One primary only	Reference^[1]^		Reference^[1]^		Reference^[1]^	
1st of 2 or more primaries	0.285 (0.271, 0.301)	<.00001	0.286 (0.271, 0.301)	<.00001	0.321 (0.305, 0.339)	<.00001
2nd of 2 or more primaries	0.893 (0.874, 0.912)	<.00001	0.836 (0.818, 0.854)	<.00001	0.862 (0.844, 0.881)	<.00001

Note: Outcome variable: Status; Cause of death recoded. Exposure variable: Sequence number. Adjust I model adjust for: sex; age; race. Adjust II model adjust for sex; age; race; origin; PRCDA; grade; laterality; diagnostic confirmation; summary stage; radiation; chemotherapy; cancer-directed surgery; regional nodes positive; marital status.

**Figure 2.**
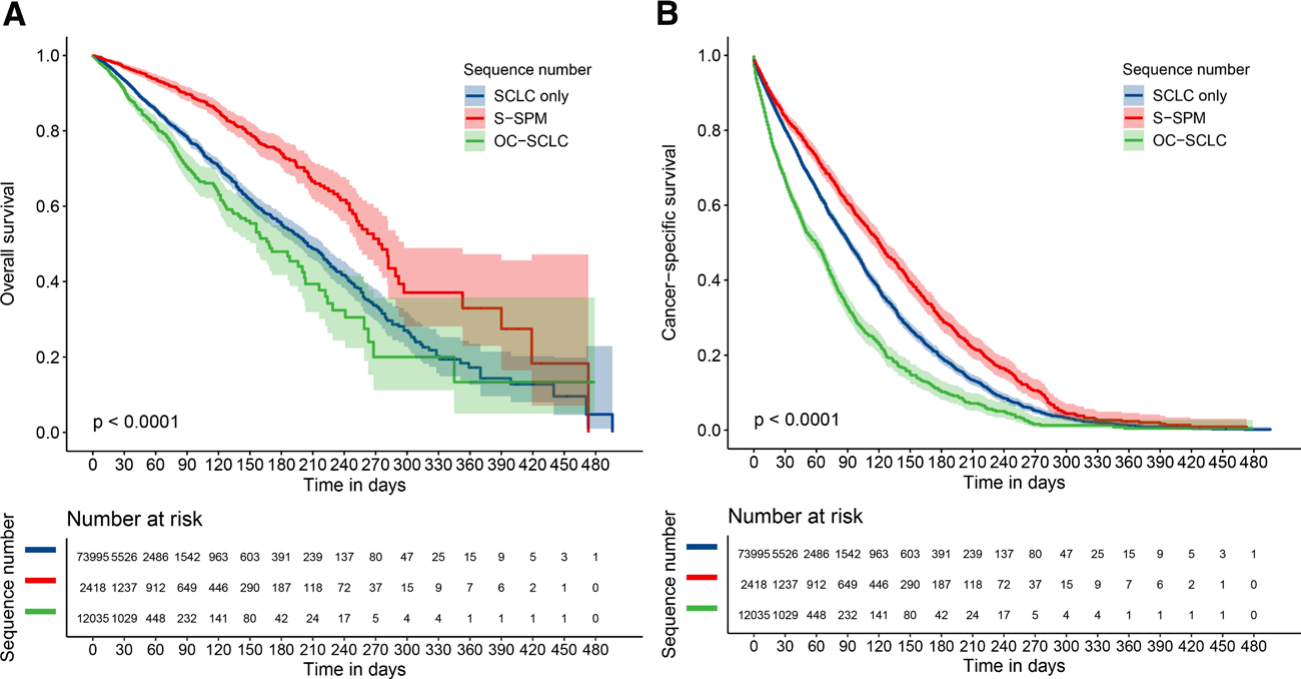
Kaplan–Meier survival curves in SCLC. (a) Overall survival curves by sequence number. (b) Cancer-specific survival curves by sequence number. SCLC = small cell lung cancer.

**Figure 3.**
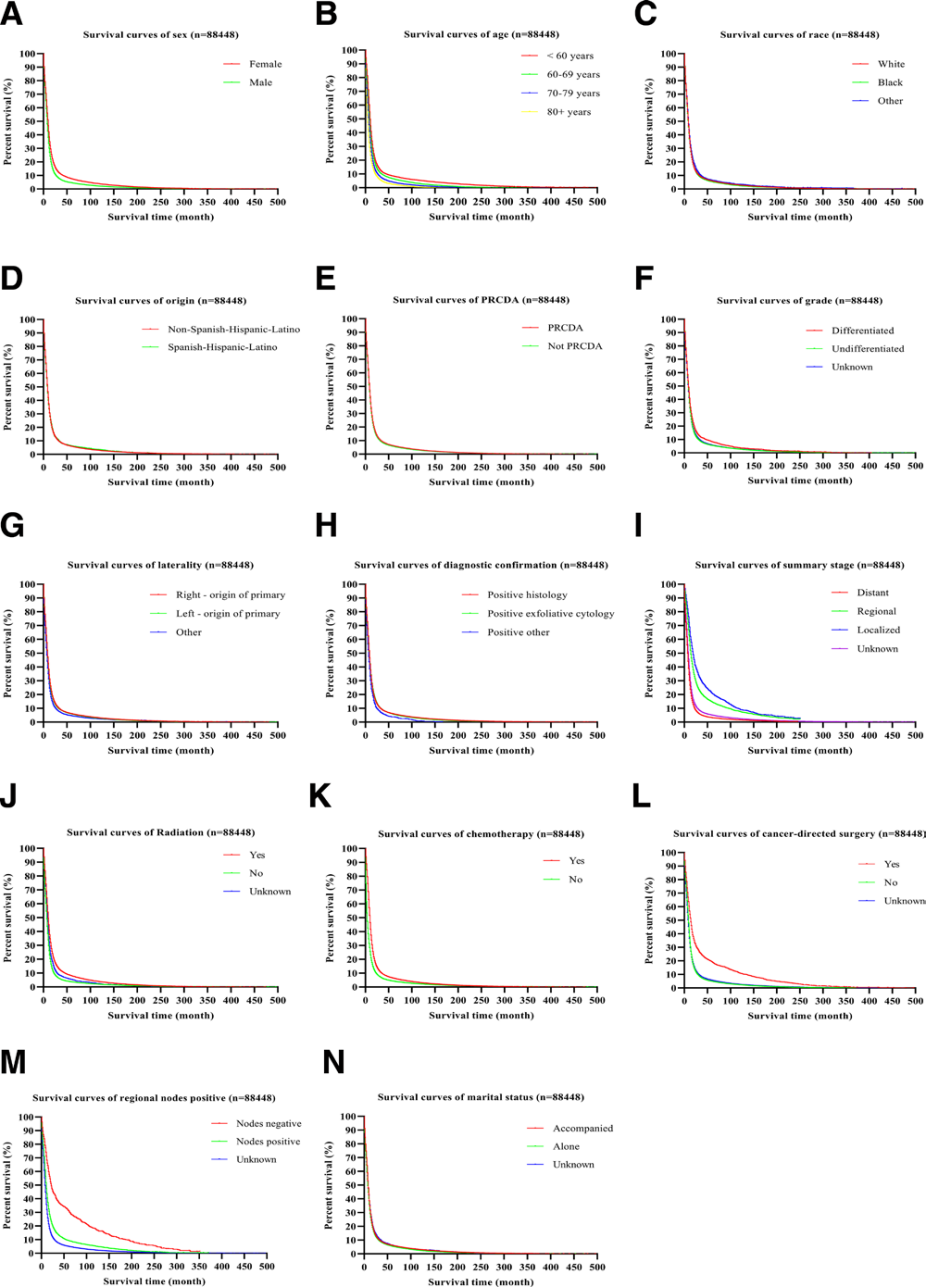
**Kaplan–Meier survival curves for SCLC covariates.** (a) According to sex. (b) According to age. (c) According to ethnicity. (d) According to origin. (e) According to PRCDA. (f) According to grade. (g) According to laterality. (h) According to confirmation. (i) According to summary stage. (j) According to radiation. (k) According to chemotherapy. (l) According to cancer-directed surgery. (m) According to regional nodes positive. (n) According to marital status. PRCDA = Purchased/Referred Care Delivery Area, SCLC = small cell lung cancer.

To further analyze the survival characteristics of patients with S-SPM, we produced Kaplan–Meier survival curves, including OS and CSS, which demonstrated that patients with S-SPM had clearly better survival than those with single-onset SCLC or OC-SCLC (Fig. 2). We also performed Kaplan–Meier survival analysis of each covariate, and covariate survival curves are shown in Figure 3.

The 88,448 patients with SCLC had a mean OS of 15.686 months (95% CI: 15.430, 15.943), the median duration of survival was 7 months (95% CI: 6.927, 7.073). The mean survival time for patients with S-SPM was 69.349 months (95% CI: 65.939, 72.759), and the median duration of survival was 34 months (95% CI: 29.900, 38.100). For patients with SCLC only, the mean duration of survival was 14.058 months (95% CI: 13.802, 14.314) and the median duration of survival was 7 months (95% CI: 6.923, 7.077). The mean survival time for patients with OC-SCLC was 14.533 months (95% CI: 13.895, 15.171), and the median duration of survival was 7 months (95% CI: 6.790, 7.210). The survival time of patients with S-SPM was significantly better than that of patients with SCLC only or OC-SCLC in terms of mean survival time and median survival time (Table 4). The survival rate for all SCLC patients was 44% at 6 months, 24% at 1 year, 15% at 18 months, 7% at 3 years, 5% at 5 years, and 2% at 10 years. The survival rate of patients with S-SPM was 78% at 6 months, 67% at 1 year, 60% at 18 months, 48% at 3 years, 39% at 5 years, and 21% at 10 years. In terms of survival at all time periods, patients with S-SPM had significantly longer survival times than the other groups (Table 5).

**Table 4:** Median and mean survival time of patients with SCLC.

			95.0%, CI					95.0%, CI		
	Mean survival time (mo)	Standard error	Lower	Upper		Median survival time (mo)	Standard error	Lower	Upper	
**Total**	15.686	0.131	15.430	15.943		7	0.037	6.927	7.073	
**Sex**										
Female	18.408	0.22	17.977	18.840		8	0.060	7.883	8.117	
Male	13.465	0.154	13.164	13.767		7	0.047	6.908	7.092	
**Age**										
<60 yrs	23.463	0.393	22.693	24.234		10	0.073	9.857	10.143	
60–69 yrs	16.176	0.19	15.803	16.548		8	0.060	7.883	8.117	
70–79 yrs	11.418	0.149	11.125	11.710		6	0.070	5.863	6.137	
80+ yrs	7.312	0.168	6.982	7.641		2	0.074	1.854	2.146	
**Race**										
White	15.586	0.137	15.318	15.855		7	0.040	6.922	7.078	
Black	15.549	0.497	14.575	16.523		7	0.129	6.747	7.253	
Other	18.528	0.893	16.777	20.279		8	0.179	7.649	8.351	
**Origin**										
Non-Spanish-Hispanic-Latino	15.675	0.132	15.416	15.934		7	0.038	6.926	7.074	
Spanish-Hispanic-Latino	15.834	0.765	14.335	17.333		7	0.221	6.566	7.434	
**PRCDA**										
PRCDA	16.223	0.215	15.802	16.644		7	0.061	6.881	7.119	
Not PRCDA	15.385	0.166	15.060	15.710		7	0.047	6.908	7.092	
**Grade**										
Differentiated	19.213	0.607	18.023		20.403	8	0.157	7.691		8.309
Undifferentiated	15.519	0.196	15.135		15.903	7	0.059	6.884		7.116
Unknown	15.398	0.184	15.037		15.758	7	0.051	6.901		7.099
**Laterality**										
Right-origin of primary	16.258	0.188	15.89		16.627	7	0.051	6.901		7.099
Left-origin of primary	15.642	0.198	15.254		16.031	7	0.058	6.887		7.113
Other	11.841	0.428	11.002		12.680	4	0.137	3.731		4.269
**Diagnostic confirmation**										
Positive histology	15.876	0.145	15.592		16.159	7	0.040	6.921		7.079
Positive exfoliative cytology	15.220	0.322	14.590		15.851	7	0.096	6.813		7.187
Positive other	9.303	0.531	8.263		10.343	4	0.289	3.434		4.566
**Summary stage**										
Distant	10.602	0.152	10.304		10.9	5	0.066	4.870		5.130
Regional	31.551	0.627	30.321		32.78	13	0.188	12.632		13.368
Localized	41.160	1.400	38.415		43.904	18	0.512	16.996		19.004
Unknown	14.588	0.152	14.290		14.886	7	0.048	6.906		7.094
**Radiation**										
Yes	21.271	0.225	20.829		21.713	10	0.056	9.89		10.11
No	10.738	0.142	10.459		11.016	4	0.055	3.892		4.108
Unknown	16.405	0.897	14.647		18.163	9	0.21	8.588		9.412
**Chemotherapy**										
Yes	18.703	0.165	18.38		19.026	9	0.038	8.925		9.075
No	8.332	0.187	7.965		8.698	1	0.026	0.95		1.05
**Cancer-directed surgery**										
Yes	39.379	1.342	36.749		42.010	13	0.365	12.285		13.715
No	14.500	0.211	14.087		14.913	8	0.072	7.858		8.142
Unknown	15.052	0.159	14.741		15.363	7	0.043	6.915		7.085
**Regional nodes positive**										
Nodes negative	59.042	2.599	53.948		64.135	21	0.939	19.161		22.839
Nodes positive	22.707	0.540	21.649		23.765	10	0.123	9.759		10.241
Unknown	14.176	0.124	13.933		14.418	7	0.039	6.924		7.076
**Marital status**										
Accompanied	16.660	0.181	16.306		17.015	8	0.047	7.907		8.093
Alone	14.349	0.194	13.969		14.729	6	0.062	5.878		6.122
Unknown	15.570	0.708	14.181		16.958	7	0.238	6.533		7.467
**Sequence number**										
One primary only	14.058	0.131	13.802		14.314	7	0.039	6.923		7.077
1st of 2 or more primaries	69.349	1.740	65.939		72.759	34	2.092	29.900		38.100
2nd of 2 or more primaries	14.533	0.326	13.895		15.171	7	0.107	6.790		7.210

SCLC = small cell lung cancer.

**Table 5:** Survival rates by time period in patients with SCLC.

	Percentage of total patients (%)	6-mo survival rate (%)	Probability density	1-yr survival rate (%)	Probability density	18-mo survival rate (%)	Probability density	3-yr survival rate (%)	Probability density	5-yr survival rate (%)	Probability density	10-yr survival rate (%)	Probability density
Total	100	44	0.045	24	0.028	15	0.012	7	0.002	5	0.001	2	0
Sex													
Female	45	48	0.042	27	0.029	18	0.013	9	0.002	6	0.001	3	0
Male	55	41	0.048	20	0.027	12	0.011	6	0.002	4	0.001	2	0
Age													
<60 yrs	24.3	55	0.05	31	0.034	20	0.016	10	0.002	8	0.001	5	0
60–69 yrs	35.3	48	0.047	25	0.031	16	0.013	8	0.002	5	0.001	2	0.001
70–79 yrs	30.4	37	0.043	19	0.024	11	0.01	5	0.002	3	0.001	1	0
80+ yrs	10.1	24	0.035	13	0.014	8	0.007	3	0.001	2	0	0	0
Race													
White	87.5	44	0.045	23	0.028	15	0.012	7	0.002	5	0.001	2	0
Black	8	44	0.046	24	0.027	15	0.012	7	0.002	4	0.001	2	0
Other	4.4	46	0.046	26	0.026	17	0.013	8	0.003	5	0.001	3	0
Origin													
Non-Spanish-Hispanic-Latino	97.3	44	0.045	24	0.028	15	0.012	7	0.002	5	0.001	2	0
Spanish-Hispanic-Latino	2.7	43	0.046	24	0.023	15	0.013	7	0.003	5	0	3	0
PRCDA													
PRCDA	36.9	45	0.046	24	0.028	15	0.012	8	0.002	5	0.001	2	0
Not PRCDA	63.1	43	0.045	23	0.028	14	0.012	7	0.002	5	0.001	2	0
Grade													
Differentiated	6.5	47	0.042	27	0.027	18	0.013	10	0.001	7	0.001	3	0.001
Undifferentiated	38.2	45	0.047	24	0.029	14	0.013	7	0.002	5	0.001	2	0
Unknown	55.3	43	0.045	23	0.027	15	0.012	7	0.002	5	0.001	2	0
Laterality													
Right-origin of primary	52.7	45	0.046	24	0.028	15	0.013	7	0.002	5	0.001	2	0
Left-origin of primary	39.9	45	0.046	24	0.029	15	0.012	7	0.002	5	0.001	2	0
Other	7.3	33	0.037	17	0.023	10	0.009	5	0.002	3	0	2	0
Diagnostic confirmation													
Positive histology	82.8	44	0.046	24	0.028	15	0.012	7	0.002	5	0.001	2	0
Positive exfoliative cytology	15.7	43	0.044	24	0.027	15	0.012	7	0.002	5	0.001	2	0
Positive other	1.5	32	0.031	16	0.019	10	0.011	4	0.001	3	0	1	0
Summary stage													
Distant	32.8	35	0.047	17	0.025	10	0.01	4	0.001	2	0	1	0
Regional	9.4	66	0.034	46	0.031	34	0.02	19	0.004	13	0.002	7	0.001
Localized	2.3	76	0.026	58	0.03	45	0.021	28	0.006	20	0.003	9	0.002
Unknown	55.5	44	0.047	22	0.029	13	0.012	6	0.002	4	0.001	2	0
Radiation													
Yes	45.7	56	0.049	33	0.033	21	0.016	11	0.003	7	0.001	3	0
No	52.6	33	0.042	15	0.024	9	0.009	4	0.001	3	0	1	0
Unknown	1.6	51	0.057	25	0.032	16	0.011	7	0.001	5	0.001	2	0.001
Chemotherapy													
Yes	70.6	54	0.055	29	0.035	18	0.015	9	0.002	6	0.001	3	0
No	29.4	20	0.023	11	0.011	7	0.005	4	0.001	3	0	1	0
Cancer-directed surgery													
Yes	3	63	0.036	46	0.025	34	0.014	23	0.004	17	0.002	10	0.001
No	24.6	46	0.046	23	0.031	14	0.013	6	0.002	4	0.001	2	0
Unknown	72.4	42	0.046	23	0.027	14	0.012	7	0.002	5	0.001	2	0
Regional nodes positive													
Nodes negative	1.4	76	0.02	62	0.022	50	0.018	36	0.004	29	0.004	16	0.003
Nodes positive	11	55	0.042	33	0.03	23	0.016	12	0.003	8	0.001	4	0.001
Unknown	87.6	42	0.046	22	0.028	13	0.012	6	0.002	4	0.001	2	0
Marital status													
Accompanied	55.6	46	0.048	25	0.029	15	0.013	8	0.002	5	0.001	3	0
Alone	41.2	41	0.042	22	0.026	14	0.011	7	0.002	4	0.001	2	0
Unknown	3.2	43	0.038	24	0.026	15	0.013	7	0.003	5	0.001	2	0
Sequence number													
One primary only	83.7	43	0.047	22	0.028	13	0.012	6	0.002	4	0.001	2	0
1st of 2 or more primaries	2.7	78	0.02	67	0.017	60	0.008	48	0.005	39	0.004	21	0.003
2nd of 2 or more primaries	13.6	43	0.043	24	0.027	15	0.012	7	0.002	4	0.001	2	0

SCLC = small cell lung cancer

## 4. Discussion

Currently, the number of patients with 2 or more malignancies is increasing. We believe that there may be several reasons for this phenomenon. The increased life expectancy of people because of better treatment for cardiovascular diseases has certainly led to a general increase in the prevalence of malignant tumors. In addition, the increased utilization of chemotherapy and radiotherapy for the first tumor might have increased the probability of genetic mutations and thus the number of secondary cancers.^[18,19]^ Previous studies have shown that as survival time increases, cancer survivors experience a higher than average risk of developing other cancers secondary to the original cancer.^[20–22]^ However, multiple primary cancer treatment options are different from those for recurrent and metastatic cancers, and often require a combination of multiple therapies, which is a challenge for health care professionals. Previous studies have addressed the incidence and survival of multiple primary cancers^[23–25]^; however, these studies had a low volume of case data and a relatively homogeneous approach. The follow-up data for small cell carcinoma in SEER was updated in April 2021; therefore, a more in-depth study of SCLC based on the most recent data is required.

Among patients with malignancy, the development of new cancers in cancer survivors is often regarded as a poor prognostic risk factor. Priante et al^[26]^ retrospectively studied 624 patients who had upper respiratory tract squamous cell carcinoma and analyzed their likelihood for progression to a second primary tumor and their survival rates. The results showed that the 5-year survival rate for this cancer secondary to a second primary tumor was only 32.2%. Patrucco et al^[27]^ demonstrated that more than half of patients with head and neck cancer (51.9%) died because of a second primary tumor, and that a second tumor significantly worsened patient prognosis and further reduced OS. However, the above conclusions might not apply to individuals with lung cancer. Duchateau et al^[28]^ demonstrated that patients with a second tumor in non-small cell lung cancer tended to experience better OS than patients without a second primary tumor. This is similar to our findings; however, that study was not conducted on patients with SCLC. There are fewer studies on patients with SCLC secondary to multiple cancers, and there is increasing concern about the therapy and prognosis for patients with S-SPM; therefore, there is a need for a more in-depth study on SCLC.

After analysis, we found that patients with S-SPM had significantly longer median survival and long-term survival than patients with SCLC only or OC-SCLC. There might be several explanations for this phenomenon. First, patients with SCLC who had developed a second cancer were generally in better health and more psychologically active, while patients with SCLC only might die prematurely because of poorer health or higher tumor malignancy; therefore, patients with S-SPM might experience longer survival than those with SCLC alone. Second, when cancer survivors of SCLC develop a second cancer, inevitably additional anti-tumor treatments are administered against the second cancer, and these subsequent treatments might simultaneously act as anti-SCLC therapies. Thirdly, patients with SCLC generally show a poor response to immune checkpoint blockade,^[29]^ and patients with SCLC alone might have a deficiency in immune surveillance, resulting in “immune escape” without activation of the immune system against SCLC. However, the second cancer in patients with S-SPM might activate not only the body’s immune mechanisms against the secondary cancer, but also the immune mechanisms associated with SCLC and act as an anti-SCLC agent.

The study by Heyne et al^[30]^ concluded that patients with SCLC who had a second primary tumor had an even higher mortality rate than patients with recurrent SCLC, which is contrary to our findings. Heyne et al only analyzed 14 eligible patients and the amount of data was so small that bias in the data analysis was inevitable. A study by Aguiló et al^[31]^ analyzed all 2030 patients diagnosed with lung cancer, including SCLC, at a local hospital between 1990 and 2004, and concluded that multiple primary cancers did not lead to a poorer prognosis. However, the data in that study were not up to date and the amount of data was small. Outdated data do not adequately represent the SCLC population, so their conclusions are controversial. In contrast, our research involved an large sample size, including 88,448 eligible patients, and we performed univariate analysis, Kaplan–Meier survival analysis, life table analysis, and multiple regression analysis with multivariate adjustment to eliminate the interference of other covariates. Thus, our study is more convincing.

Our research has some limitations. First, this was a retrospective analysis, and further clinical and basic studies are needed to confirm our results. Second, although 27.8% of the U.S. population is already covered by the SEER database, our research would have been more convincing if we had access to more data, such as the entire world’s SCLC data.

## 5. Conclusions

By analyzing 88,448 patients with SCLC in the SEER database, we found that the duration of survival was clearly superior for patients with S-SPM than for patients with SCLC only or OC-SCLC. We also noted that the occurrence of SPM in patients with SCLC did not necessarily lead to a poor prognosis. When patients with SCLC develop a second cancer during survival, they should receive more aggressive treatment and should not give up treatment easily. The findings of the present study might provide valuable insights into the ongoing monitoring of cancer survivors with combined SPM in SCLC.

## Acknowledgments

We would like to gratefully acknowledge the time and effort of the participants during the data collection phase of the SEER project. We are equally grateful to the National Natural Science Foundation of China (81860379 and 82160410) for supporting this study.

## Author contributions

**Conceptualization:** Silin Wang, Shengfei Huang, Lang Su, Jiayue Ye, Yiping Wei.

**Data curation:** Silin Wang, Sheng Hu, Yiping Wei.

**Formal analysis:** Silin Wang, Sheng Hu, Yang Zhang.

**Funding acquisition:** Yiping Wei.

**Investigation:** Silin Wang, Sheng Hu.

**Methodology:** Qiang Guo, Deyuan Zhang, Wenxiong Zhang.

**Project administration:** Wenxiong Zhang.

**Resources:** Silin Wang, Shengfei Huang.

**Software:** Bo Wu.

**Supervision:** Wenxiong Zhang.

**Writing – original draft:** Silin Wang, Sheng Hu.

**Writing – review & editing:** Yiping Wei.

## Supplementary Material


