# Supplementary material for: Better survival and prognosis in SCLC survivors after combined second primary malignancies: A SEER database-based study
Source: Medicine (Baltimore). 2023 Feb 10;102(6):e32772. doi: 10.1097/MD.0000000000032772 (PMC9907942; doi:10.1097/MD.0000000000032772)
Supplement: Supplementary file 1 [file medi-102-e32772-s001.pdf]

Table S1. Stratified analysis of small cell lung cancer.

Sub-group	N	Status	P-value	Cause of death recoded	P-value
		HR (95%CI)		HR (95%CI)	
Year of diagnosis Tertile					
Low					
One primary only	25045	Reference(1)		Reference(1)	
1st of 2 or more primaries	743	0.404 (0.375, 0.435)	<0.0001	0.313 (0.286, 0.343)	<0.0001
2nd of 2 or more primaries	2413	0.968 (0.928, 1.009)	0.1261	0.894 (0.854, 0.935)	<0.0001
Middle					
One primary only	24005	Reference(1)		Reference(1)	
1st of 2 or more primaries	820	0.344 (0.320, 0.369)	<0.0001	0.259 (0.237, 0.284)	<0.0001
2nd of 2 or more primaries	3898	1.009 (0.975, 1.044)	0.6130	0.937 (0.903, 0.972)	0.0005
High					
One primary only	24945	Reference(1)		Reference(1)	
1st of 2 or more primaries	855	0.363 (0.336, 0.393)	<0.0001	0.294 (0.269, 0.322)	<0.0001
2nd of 2 or more primaries	5724	0.980 (0.951, 1.010)	0.1990	0.887 (0.859, 0.917)	<0.0001
Sex					
Female					
One primary only	32613	Reference(1)		Reference(1)	
1st of 2 or more primaries	1091	0.351 (0.329, 0.375)	<0.0001	0.263 (0.242, 0.285)	<0.0001
2nd of 2 or more primaries	6057	0.973 (0.946, 1.001)	0.0569	0.891 (0.864, 0.919)	<0.0001
Male					
One primary only	41382	Reference(1)		Reference(1)	
1st of 2 or more primaries	1327	0.379 (0.358, 0.402)	<0.0001	0.304 (0.284, 0.326)	<0.0001
2nd of 2 or more primaries	5978	0.996 (0.969, 1.024)	0.7762	0.914 (0.887, 0.942)	<0.0001
Age					
< 60 years					
One primary only	19272	Reference(1)		Reference(1)	
1st of 2 or more primaries	642	0.326 (0.299, 0.355)	<0.0001	0.243 (0.218, 0.270)	<0.0001
2nd of 2 or more primaries	1543	0.881 (0.835, 0.929)	<0.0001	0.802 (0.757, 0.849)	<0.0001
60-69 years					
One primary only	26472	Reference(1)		Reference(1)	
1st of 2 or more primaries	964	0.353 (0.330, 0.378)	<0.0001	0.274 (0.252, 0.298)	<0.0001
2nd of 2 or more primaries	3780	0.879 (0.849, 0.911)	<0.0001	0.816 (0.785, 0.847)	<0.0001
70-79 years					
One primary only	21464	Reference(1)		Reference(1)	
1st of 2 or more primaries	635	0.413 (0.380, 0.448)	<0.0001	0.333 (0.301, 0.368)	<0.0001
2nd of 2 or more primaries	4784	0.927 (0.898, 0.957)	<0.0001	0.857 (0.828, 0.887)	<0.0001
80+ years					
One primary only	6787	Reference(1)		Reference(1)	
1st of 2 or more primaries	177	0.479 (0.411, 0.560)	<0.0001	0.373 (0.310, 0.450)	<0.0001

Continued Table S1

Sub-group	N	Status	P-value	Cause of death recoded	P-value
		HR (95%CI)		HR (95%CI)	
2nd of 2 or more primaries	1928	0.911 (0.866, 0.959)	0.0004	0.837 (0.792, 0.885)	<0.0001
Race					
White					
One primary only	64636	Reference(1)		Reference(1)	
1st of 2 or more primaries	2089	0.362 (0.345, 0.379)	<0.0001	0.277 (0.262, 0.294)	<0.0001
2nd of 2 or more primaries	10692	0.973 (0.953, 0.994)	0.0108	0.896 (0.876, 0.916)	<0.0001
Black					
One primary only	5964	Reference(1)		Reference(1)	
1st of 2 or more primaries	209	0.425 (0.367, 0.492)	<0.0001	0.357 (0.301, 0.424)	<0.0001
2nd of 2 or more primaries	929	0.928 (0.865, 0.997)	0.0405	0.821 (0.759, 0.888)	<0.0001
Other					
One primary only	3395	Reference(1)		Reference(1)	
1st of 2 or more primaries	120	0.369 (0.302, 0.452)	<0.0001	0.320 (0.253, 0.406)	<0.0001
2nd of 2 or more primaries	414	1.047 (0.943, 1.163)	0.3854	0.939 (0.837, 1.054)	0.2871
Origin					
Non-Spanish-Hispanic-Latino					
One primary only	71936	Reference(1)		Reference(1)	
1st of 2 or more primaries	2349	0.367 (0.352, 0.384)	<0.0001	0.286 (0.271, 0.302)	<0.0001
2nd of 2 or more primaries	11732	0.975 (0.956, 0.995)	0.0135	0.895 (0.876, 0.914)	<0.0001
Spanish-Hispanic-Latino					
One primary only	2059	Reference(1)		Reference(1)	
1st of 2 or more primaries	69	0.351 (0.270, 0.456)	<0.0001	0.257 (0.185, 0.357)	<0.0001
2nd of 2 or more primaries	303	0.896 (0.790, 1.016)	0.0862	0.802 (0.699, 0.920)	0.0017
PRCDA					
PRCDA					
One primary only	27193	Reference(1)		Reference(1)	
1st of 2 or more primaries	871	0.360 (0.335, 0.387)	<0.0001	0.269 (0.246, 0.295)	<0.0001
2nd of 2 or more primaries	4575	0.971 (0.940, 1.002)	0.0697	0.893 (0.863, 0.925)	<0.0001
Not PRCDA					
One primary only	46802	Reference(1)		Reference(1)	
1st of 2 or more primaries	1547	0.370 (0.351, 0.391)	<0.0001	0.295 (0.276, 0.314)	<0.0001
2nd of 2 or more primaries	7460	0.975 (0.951, 1.000)	0.0486	0.893 (0.869, 0.917)	<0.0001
Grade					
Differentiated					
One primary only	4715	Reference(1)		Reference(1)	
1st of 2 or more primaries	202	0.352 (0.301, 0.411)	<0.0001	0.280 (0.232, 0.337)	<0.0001
2nd of 2 or more primaries	854	0.908 (0.843, 0.978)	0.0113	0.839 (0.775, 0.910)	<0.0001
Undifferentiated					

Continued Table S1

Sub-group	N	Status HR (95%CI)	P-value	Cause of death recoded HR (95%CI)	P-value
One primary only	28802	Reference(1)		Reference(1)	
1st of 2 or more primaries	964	0.360 (0.337, 0.385)	<0.0001	0.283 (0.261, 0.307)	<0.0001
2nd of 2 or more primaries	4017	0.991 (0.959, 1.025)	0.5992	0.912 (0.879, 0.945)	<0.0001
Unknown					
One primary only	40478	Reference(1)		Reference(1)	
1st of 2 or more primaries	1252	0.375 (0.353, 0.399)	<0.0001	0.289 (0.268, 0.311)	<0.0001
2nd of 2 or more primaries	7164	0.971 (0.946, 0.997)	0.0273	0.888 (0.864, 0.914)	<0.0001
Laterality					
Right - origin of primary					
One primary only	38976	Reference(1)		Reference(1)	
1st of 2 or more primaries	1304	0.370 (0.349, 0.393)	<0.0001	0.284 (0.264, 0.305)	<0.0001
2nd of 2 or more primaries	6342	0.960 (0.934, 0.986)	0.0033	0.886 (0.860, 0.913)	<0.0001
Left - origin of primary					
One primary only	29645	Reference(1)		Reference(1)	
1st of 2 or more primaries	988	0.373 (0.349, 0.399)	<0.0001	0.301 (0.278, 0.326)	<0.0001
2nd of 2 or more primaries	4697	0.985 (0.955, 1.017)	0.3610	0.906 (0.875, 0.937)	<0.0001
Other					
One primary only	5374	Reference(1)		Reference(1)	
1st of 2 or more primaries	126	0.307 (0.253, 0.372)	<0.0001	0.205 (0.159, 0.264)	<0.0001
2nd of 2 or more primaries	996	0.973 (0.909, 1.043)	0.4416	0.845 (0.783, 0.912)	<0.0001
Diagnostic confirmation					
Positive histology					
One primary only	61372	Reference(1)		Reference(1)	
1st of 2 or more primaries	2039	0.371 (0.354, 0.388)	<0.0001	0.289 (0.273, 0.306)	<0.0001
2nd of 2 or more primaries	9827	0.974 (0.953, 0.996)	0.0198	0.893 (0.872, 0.914)	<0.0001
Positive exfoliative cytology					
One primary only	11515	Reference(1)		Reference(1)	
1st of 2 or more primaries	361	0.350 (0.312, 0.392)	<0.0001	0.268 (0.233, 0.309)	<0.0001
2nd of 2 or more primaries	2038	0.969 (0.923, 1.017)	0.1987	0.893 (0.848, 0.941)	<0.0001
Positive other					
One primary only	1108	Reference(1)		Reference(1)	
1st of 2 or more primaries	18	0.346 (0.211, 0.570)	<0.0001	0.287 (0.157, 0.524)	<0.0001
2nd of 2 or more primaries	170	0.951 (0.807, 1.122)	0.5533	0.874 (0.732, 1.043)	0.1346
Summary stage					
Distant					
One primary only	23763	Reference(1)		Reference(1)	
1st of 2 or more primaries	489	0.383 (0.348, 0.422)	<0.0001	0.319 (0.286, 0.357)	<0.0001
2nd of 2 or more primaries	4724	1.054 (1.022, 1.088)	0.0010	0.960 (0.928, 0.993)	0.0169

Continued Table S1

Sub-group	N	Status HR (95%CI)	P-value	Cause of death recoded HR (95%CI)	P-value
Regional					
One primary only	6386	Reference(1)		Reference(1)	
1st of 2 or more primaries	453	0.421 (0.379, 0.468)	<0.0001	0.326 (0.286, 0.371)	<0.0001
2nd of 2 or more primaries	1486	1.036 (0.976, 1.099)	0.2492	0.950 (0.890, 1.014)	0.1217
Localized					
One primary only	1387	Reference(1)		Reference(1)	
1st of 2 or more primaries	159	0.400 (0.330, 0.485)	<0.0001	0.325 (0.255, 0.415)	<0.0001
2nd of 2 or more primaries	509	0.992 (0.890, 1.106)	0.8829	0.880 (0.776, 0.997)	0.0454
Unknown					
One primary only	42459	Reference(1)		Reference(1)	
1st of 2 or more primaries	1317	0.380 (0.359, 0.402)	<0.0001	0.292 (0.272, 0.313)	<0.0001
2nd of 2 or more primaries	5316	0.967 (0.940, 0.996)	0.0256	0.899 (0.871, 0.927)	<0.0001
Radiation					
Yes					
One primary only	34134	Reference(1)		Reference(1)	
1st of 2 or more primaries	1400	0.353 (0.334, 0.374)	<0.0001	0.274 (0.255, 0.294)	<0.0001
2nd of 2 or more primaries	4922	0.930 (0.902, 0.959)	<0.0001	0.854 (0.826, 0.883)	<0.0001
No					
One primary only	38649	Reference(1)		Reference(1)	
1st of 2 or more primaries	968	0.408 (0.381, 0.436)	<0.0001	0.320 (0.296, 0.347)	<0.0001
2nd of 2 or more primaries	6945	0.959 (0.935, 0.985)	0.0017	0.879 (0.855, 0.904)	<0.0001
Unknown					
One primary only	1212	Reference(1)		Reference(1)	
1st of 2 or more primaries	50	0.338 (0.250, 0.458)	<0.0001	0.273 (0.189, 0.395)	<0.0001
2nd of 2 or more primaries	168	1.031 (0.876, 1.214)	0.7117	0.956 (0.801, 1.140)	0.6143
Chemotherapy					
Yes					
One primary only	52389	Reference(1)		Reference(1)	
1st of 2 or more primaries	1908	0.358 (0.341, 0.376)	<0.0001	0.281 (0.265, 0.299)	<0.0001
2nd of 2 or more primaries	8114	0.952 (0.929, 0.975)	<0.0001	0.879 (0.857, 0.902)	<0.0001
No					
One primary only	21606	Reference(1)		Reference(1)	
1st of 2 or more primaries	510	0.422 (0.385, 0.462)	<0.0001	0.319 (0.285, 0.357)	<0.0001
2nd of 2 or more primaries	3921	0.950 (0.918, 0.983)	0.0035	0.859 (0.828, 0.892)	<0.0001
Cancer-directed surgery					
Yes					
One primary only	2146	Reference(1)		Reference(1)	
1st of 2 or more primaries	170	0.443 (0.378, 0.519)	<0.0001	0.341 (0.277, 0.419)	<0.0001

Continued Table S1

Sub-group	N	Status	P-value	Cause of death recoded	P-value
		HR (95%CI)		HR (95%CI)	
2nd of 2 or more primaries	308	0.878 (0.778, 0.990)	0.0330	0.792 (0.690, 0.910)	0.0010
No					
One primary only	18495	Reference(1)		Reference(1)	
1st of 2 or more primaries	576	0.356 (0.326, 0.387)	<0.0001	0.271 (0.244, 0.302)	<0.0001
2nd of 2 or more primaries	2719	1.014 (0.974, 1.056)	0.4982	0.947 (0.907, 0.989)	0.0138
Unknown					
One primary only	53354	Reference(1)		Reference(1)	
1st of 2 or more primaries	1672	0.369 (0.350, 0.389)	<0.0001	0.291 (0.273, 0.310)	<0.0001
2nd of 2 or more primaries	9008	0.964 (0.942, 0.986)	0.0017	0.879 (0.857, 0.901)	<0.0001
Regional nodes positive					
Nodes negative					
One primary only	881	Reference(1)		Reference(1)	
1st of 2 or more primaries	131	0.457 (0.369, 0.565)	<0.0001	0.385 (0.295, 0.504)	<0.0001
2nd of 2 or more primaries	254	0.980 (0.839, 1.146)	0.8011	0.856 (0.713, 1.027)	0.0945
Nodes positive					
One primary only	7766	Reference(1)		Reference(1)	
1st of 2 or more primaries	340	0.386 (0.342, 0.435)	<0.0001	0.295 (0.255, 0.342)	<0.0001
2nd of 2 or more primaries	1616	0.975 (0.921, 1.031)	0.3716	0.889 (0.836, 0.945)	0.0002
Unknown					
One primary only	65348	Reference(1)		Reference(1)	
1st of 2 or more primaries	1947	0.372 (0.355, 0.390)	<0.0001	0.291 (0.274, 0.308)	<0.0001
2nd of 2 or more primaries	10165	0.997 (0.976, 1.018)	0.7575	0.916 (0.895, 0.937)	<0.0001
Marital status					
Accompanied					
One primary only	41184	Reference(1)		Reference(1)	
1st of 2 or more primaries	1460	0.363 (0.343, 0.384)	<0.0001	0.284 (0.266, 0.304)	<0.0001
2nd of 2 or more primaries	6521	0.986 (0.960, 1.012)	0.2858	0.904 (0.878, 0.930)	<0.0001
Alone					
One primary only	30461	Reference(1)		Reference(1)	
1st of 2 or more primaries	900	0.374 (0.349, 0.402)	<0.0001	0.288 (0.264, 0.315)	<0.0001
2nd of 2 or more primaries	5091	0.959 (0.930, 0.988)	0.0065	0.879 (0.851, 0.908)	<0.0001
Unknown					
One primary only	2350	Reference(1)		Reference(1)	
1st of 2 or more primaries	58	0.377 (0.284, 0.502)	<0.0001	0.277 (0.193, 0.398)	<0.0001
2nd of 2 or more primaries	423	0.912 (0.819, 1.016)	0.0952	0.849 (0.756, 0.954)	0.0061

Note: Outcome variable: Status; Cause of death recoded. Exposure variable: Sequence number.
